# Chronometabolism: The Timing of the Consumption of Meals Has a Greater Influence Than Glycemic Index (GI) on the Postprandial Metabolome

**DOI:** 10.3390/metabo13040490

**Published:** 2023-03-29

**Authors:** Yi Ning Yong, Jiangwen Dong, Leroy Sivappiragasam Pakkiri, Christiani Jeyakumar Henry, Sumanto Haldar, Chester Lee Drum

**Affiliations:** 1 Clinical Nutrition Research Centre (CNRC), Singapore Institute of Food and Biotechnology Innovation (SIFBI), Agency for Science, Technology and Research (A*STAR), 14 Medical Drive, MD6 #07-02, Singapore 117599, Singapore; 2 Cardiovascular Research Institute (CVRI), National University Health System (NUHS), 14 Medical Drive, MD6 Level 8, Singapore 117599, Singapore; 3 Department of Medicine, Yong Loo Lin School of Medicine, National University of Singapore (NUS), Singapore 119228, Singapore; 4 Department of Biochemistry, National University of Singapore (NUS), 8 Medical Drive, MD7, Singapore 117596, Singapore

**Keywords:** glycemic index, timing of meal intake, metabolomics, glucose homeostasis, cardiometabolic disease risk

## Abstract

Eating late in the day is associated with circadian desynchrony, resulting in dysregulated metabolism and increased cardiometabolic disease risk. However, the underlying mechanisms remain unclear. Using targeted metabolomics of postprandial plasma samples from a secondary analysis of a randomised 2 × 2 crossover study in 36 healthy older Chinese adults, we have compared postprandial metabolic responses between high (HI) glycemic index (GI) or low-GI (LO) meals, consumed either at breakfast (BR) or at dinner (DI). 29 out of 234 plasma metabolites exhibited significant differences (*p* < 0.05) in postprandial AUC between BR and DI sessions, whereas only five metabolites were significantly different between HI and LO sessions. There were no significant interactions between intake timing and meal GI. Lower glutamine: glutamate ratio, lower lysine and higher trimethyllysine (TML) levels were found during DI compared with BR, along with greater postprandial reductions (δAUC) in creatine and ornithine levels during DI, indicating a worse metabolic state during the evening DI period. Greater reductions (δAUC) in postprandial creatine and ornithine were also observed during HI compared with LO (both *p* < 0.05). These metabolomic changes may indicate potential molecular signatures and/or pathways linking metabolic responses with cardiometabolic disease risk between different meal intake timings and/or meals with variable GI.

## 1. Introduction

Dysregulated postprandial glycemia is an important risk factor in the aetiology of cardiometabolic diseases, including type 2 diabetes [1]. The quantity and quality of carbohydrates in the diet play a pivotal role in the development of postprandial hyperglycemia, with the glycemic index (GI) of foods applied extensively as a global indicator of the glycemic potential of foods/meals [2]. There are several mechanisms which associate dysregulated glycemia with the pathogenesis of cardiometabolic disease, including a plethora of metabolic perturbations, such as inflammation, oxidative stress, excess protein glycation, endothelial dysfunction and insulin resistance. Hyperglycemia and the formation of advanced glycated end products (AGEs) elicit oxidative stress and nitrosative stress through multiple modes of action, such as free radical production and inhibition of the cell/tissue’s own antioxidant defence mechanism [3,4]. The increased availability of glucose during the postprandial state results in greater production of reactive oxygen species (ROS) and the activation of various stress responses, which impedes the insulin receptor pathway, leading to the development of insulin resistance in tissues [5].

Besides the consideration of GI, the timing of intake also plays an integral role in the management of metabolic homeostasis. From an evolutionary point of view, as food intake and energy expenditure are anticipated earlier during the day while energy conservation and rest are prioritised at night [6], the consumption of carbohydrate-rich foods at night per se is associated with impaired glucose homeostasis. Thus, repeated and prolonged night-time feeding may lead to the development of circadian desynchrony, a phenomenon whereby the peripheral clocks and central clock governing the sleep-wake cycle are misaligned, progressively increasing the risk of developing metabolic syndrome, insulin resistance and cardiometabolic diseases [7]. Presently, while the contribution of circadian misalignment to increased disease risk has been extensively illustrated using in vitro and animal studies, there remain gaps in our current understanding of the underlying mechanisms in humans, particularly how key metabolic pathways interact with the timing of food consumption.

Under regular conditions, up to 64% of metabolites in human plasma have been identified to exhibit significant diurnal rhythmicity, including those involved in the redox homeostasis [8]. Circadian disturbances induced by night shifts—characterised by a combination of the night eating and sleeping during the day—have been associated with increased expression of oxidative stress markers [9], while feeding exclusively during an early time window (08:00–14:00) had been shown to reduce oxidative stress in men with prediabetes [10]. While there has been wide coverage on the impacts of sleep parameters and deprivation on metabolites linking to the oxidative stress [11], there is a paucity of studies investigating the combined impact of intake timing and diet quality—which are both potent modulators of peripheral circadian rhythms governing the human metabolome [12,13]. Hence, this presents an impetus to investigate whether the plasma status of certain metabolites (including specific amino acids and their metabolic by-products) that have the potential to influence or regulate glycemic response is different between early and late feeding times to the same meals, so as to better understand the link between circadian desynchrony, dysregulated glycemia and cardiometabolic disease risk. 

We have previously reported findings from a 2 × 2 acute randomised crossover study where we assessed the interactions of low or high GI (matched for available carbohydrates) with the timing of food intake (breakfast or dinner) on glucose homeostasis, which demonstrated that dinner meal intake produced significantly worse postprandial glucose homeostasis as compared to breakfast intake, irrespective of GI [14]. In this secondary analysis, we have further investigated targeted plasma metabolites across the three-hour postprandial period in order to investigate potential mechanisms which may link glycemic perturbations associated with late consumption of foods to further increase cardiometabolic disease risk. 

## 2. Materials and Methods

### 2.1. Study Design

The study protocol and details of the recruitment process had been reported in a previous publication, [14], and approved by a Domain Specific Research Board (DSRB) ethics committee, Singapore (Reference: C/2016/00613), conducted in line with the Declaration of Helsinki 1983 and the Singapore Good Clinical Practice Guidelines. In summary, the study was undertaken in 22 men and 15 postmenopausal women, with one male participant having missing data. Hence the results reported here were for 36 participants. Participants were of Chinese ethnicity, aged (mean ± SD) 57.6 ± 4.68 years, with a BMI of 22.3 ± 1.69 kg/m^2^.

This study was designed as a 2 × 2 acute, randomised, crossover study to facilitate comparisons between (1) high-GI (GI = 92) versus low-GI (GI = 55) meals; consumed either at (2) breakfast (09:00) or at dinner (18:30), with separate sessions allocated to each volunteer in a randomised order. In brief, the low and high GI test meals (TMs) were prepared using basmati and glutinous rice, respectively, as the main carbohydrate component in each meal (each matched for approximately 75 g of available carbohydrates across all four intervention groups). Each intervention session was spaced apart by a washout period of at least three days. The same standardised meal (SM) was also provided as the meal preceding the TMs—i.e., SM dinner before the TM breakfast; or SM lunch before the TM dinner—to minimise variability between baseline metabolite levels attributed to carryover effects from each individual’s previous meal occasion. Details of the TMs and SM and their respective caloric and macronutrient contents are documented in Supplementary Table S2.

During the breakfast session, volunteers were instructed to fast overnight for 13 h following the consumption of the SM dinner before attending the study visit. Baseline blood samples (0 min) were obtained via intravenous cannulation at approximately 08:30, following which volunteers were assigned either a high-GI or low-GI breakfast (HI-BR and LO-BR, respectively) at 09:00. Blood samples were subsequently drawn at regular intervals over a period of three hours, at 30, 60, 120 and 180 min. A similar protocol was also employed for the dinner session, in which volunteers consumed the SM lunch at 12:30 and had their baseline blood samples obtained at 18:00 (amounting to a 6-h fast) before the intake of either a high-GI or low-GI dinner (HI-DI and LO-DI, respectively) at 18:30.

### 2.2. Blood Sample Collection

Blood samples for metabolomics profiling were collected in K2 EDTA plasma vacutainer tubes (BD, USA) and stored on ice immediately upon collection and centrifuged at 1500× *g* for 10 min at 4 °C within 30 min of collection. Plasma aliquots were stored at −80 °C after processing and thawed subsequently for semi-targeted metabolomics analysis via LC-MS/MS.

### 2.3. Sample Measurement

The blood samples underwent extraction using a 96-well plate according to our fixed protocol. To 50 µL of plasma, 50 µL of the standard internal mixture (3–5 µg/L of stable isotopes) was added. To this, ice-cold 360 µL Acetonitrile with 0.1% formic acid was added. The resultant mixture was then shaken for 10 min with the Agilent Bravo automated liquid handling platform set at 1000 rpm/min. Following this, the mixture was centrifuged down at 2270× *g* for 50 min at 4 °C. 150 µL of the supernatant was then transferred to a 96-microwell plate before being loaded into the auto-sampler of the Agilent 1290 Infinity LC-6495C triple quadrupole (QqQ) MS/MS system (Agilent Technologies, Santa Clara, CA, USA).

A peek coated SeQuant^®^ZIC^®^-cHILIC 3 µm, 100 Å 100 × 2.1 mm HPLC column (Merck Pte Ltd., Singapore) with zwitterionic phosphorylcholine head groups was utilised. The organic solvent was Acetonitrile with 0.1% Formic Acid (Solvent A), and the aqueous solvent was 20 mM Ammonium Format pH 4.5 (Solvent B). The percentage of each solvent used at each time was varied using the Binary pump (Agilent Model G7120A) with the percentage of Solvent B as follows: 10% at 0.00 min, 70% between 9:00 to 11:10 min, and 10% between 11:.10 to 11:50 min. The sample injection volume was 10 µL, and the flow rate of the solvent system was 0.400 mL/min.

2-HPLC column with switching function using the Agilent Quick-Change valve head, 2-position/10-port (Cat. No: 5067-4240), was utilised to reduce the assay cycle time. Dynamic Multiple Reaction Monitoring (MRM) was used with a retention time (RT) delta window of 1.2–1.5 min. For time filtering, the peak width is set to 0.03 min, and the Delta EMV (+) and (−) are set at 200. Electrospray ionisation was used in both positive and negative ionisation modes, with the following source parameters: the gas temperature at 290 °C, gas flow of 20 L/min, nebuliser pressure at 35 psi, sheath gas temperature at 350 °C, sheath gas flow of 12 L/min, the capillary voltage of 4000 V, nozzle voltage of 500 V, and chamber current of 0.20 µA. The iFunnel parameters had a high-pressure RF of +200 V to −90 V and a low-pressure RF of +100 V to −60 V. Analytes were normalised to Internal standard (ISTD) peak height [15] (Supplementary Methods, Supplementary Table S1). Acquisitions were performed using the Agilent MassHunter Workstation Acquisition (10.0.127).

### 2.4. Data Generation and Quality Control

**Figure 1:**
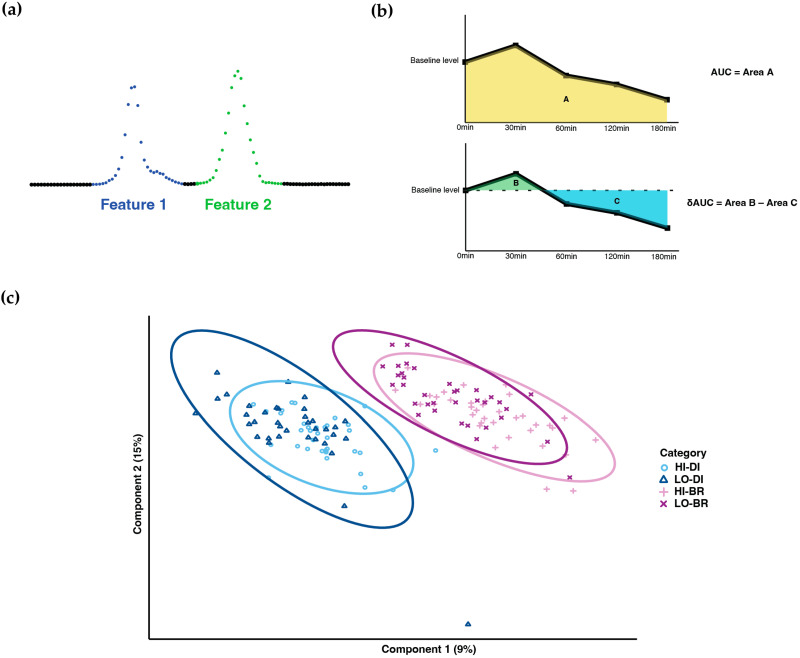
Metabolome profiling. (**a**) Naming methodology of metabolites with multiple peaks. (**b**) Calculation of AUC and δAUC. (**c**) PLS-DA result of sample clustering (HI-DI: high-GI dinner; LO-DI: low-GI dinner; HI-BR: high-GI breakfast; LO-BR: low-GI breakfast).

Chromatographic peak integration and batch correction [15] were performed to generate the initial data for statistical analysis. In the ion-chromatograms, some transitions exhibited multiple peaks in the expected RT window; we named the first peak with the smallest RT as metabolite feature 1, the second peak as metabolite feature 2, and so on (Figure 1a). These features were then treated equally as single metabolites during statistical analysis.

Low-quality analytes were filtered using the Coefficient of Variation (CoV) and dispersion ratio (D-Ratio) (Supplementary Methods) [16]. Metabolites with CoV > 30% and D-Ratio > 50% were filtered out. Ion-chromatograms of key metabolites identified in statistical analysis were all manually reviewed for peak shape and signal strength.

### 2.5. Statistical Analysis

Statistical analysis was performed using R 4.1.1. The area under the curve (AUC) and baseline-corrected area under the curve (δAUC) of 4 TMs were calculated for each metabolite and subject to represent the post-prandial metabolic responses: As shown in Figure 1b, area A was calculated as AUC. With respect to the baseline level at 0 min, area B is the incremental AUC, area C is the decremental AUC, and δAUC was calculated as area B minus area C. Based on the AUC values, PLS-DA was performed to cluster the samples.

Linear mixed model (LMM) and general linear model (GLM) were applied to compare the AUC and δAUC difference in high-GI (HI) vs. low-GI (LO) and dinner (DI) vs. breakfast (BR):

(1) (δ)AUC~GI+Time+GI:Time+(1|Subject)

where (δ)AUC is the AUC or δAUC values, GI is the TM GI content (high or low GI), Time is the TM timing (dinner or breakfast), GI:Time is the interaction term of GI and Time. In comparing AUC differences, the random effect term (1|Subject) was also used to adjust for repeated measurements of subjects. 

For several key metabolites, the metabolite precursor-product ratios were also calculated. The same analysis process was performed to compare the ratio difference under different scenarios.

All the *p*-values were adjusted by Benjamini–Hochberg method [17] for multiple comparisons, *p*-value cut-off was set at 0.05.

## 3. Results

### 3.1. Metabolome Profiling

321 metabolites were identified and measured by LC-MS/MS assay (Supplementary Table S1). 256 metabolites passed the initial quality filtering by CoV and D-Ratio. After a manual review of the chromatographic response, 234 metabolites remained.

After PLS-DA, samples showed clear clustering between dinner and breakfast, whereas most overlapped between high-GI and low-GI TM (Figure 1c).

### 3.2. Differences in Metabolites and Related Pathway under Different GI Content and TM Timing

**Figure 2:**
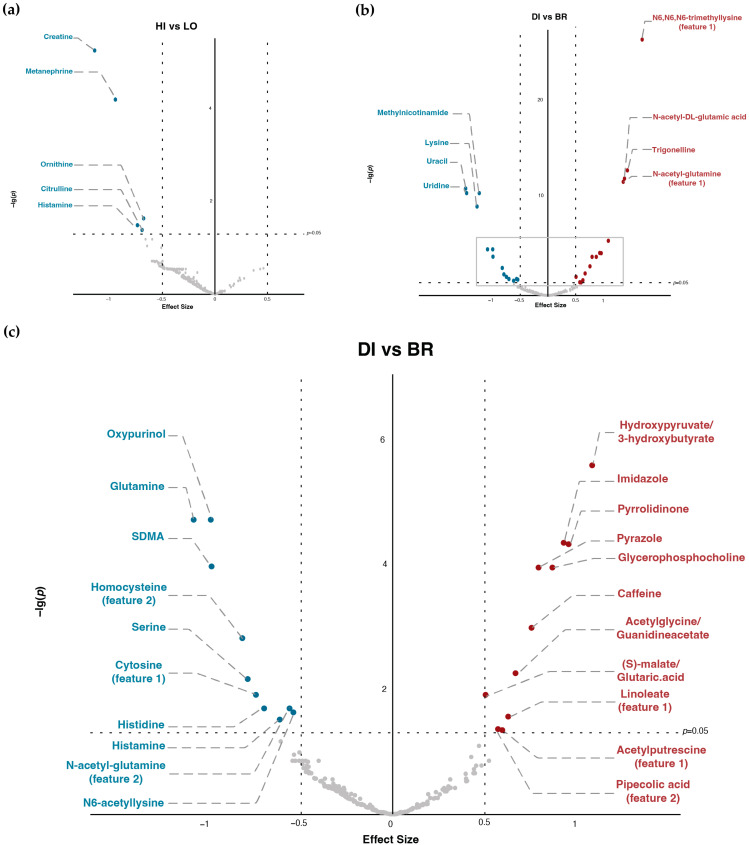
Alteration of metabolites AUC under different TM timing and GI content. (**a**) Volcano plot of high-GI versus low-GI TM (HI: high-GI; LO: low-GI; effect size: AUC_(HI)_–AUC_(LO)_). (**b**) Volcano plot of dinner versus breakfast (DI: dinner; BR: breakfast; effect size: AUC_(DI)_–AUC_(BR)_; figure area within the grey frame is zoomed in as (**c**)). (**c**) Zoomed-in volcano plot of dinner versus breakfast (DI: dinner; BR: breakfast; effect size: AUC_(DI)_–AUC_(BR)_).

As shown in Figure 2, Table 1 and Table 2, there were significant differences in the AUC of 29 metabolites for DI vs. BR and five metabolites for HI vs. LO, whereas, in δAUC, only creatine and ornithine had significant differences for DI vs. BR and HI vs. LO. In addition, we did not observe significant interactions between GI and Time for either AUC or δAUC comparisons in any of the metabolites.

### 3.3. Top Metabolites and Pathways Identified by (δ)AUC Difference of Overall Comparison

Results indicated that many metabolites with significant *p*-values were involved in the Arginine-NO pathway (Table 1 and Table 2, Supplementary Figure S2). Comparing DI versus BR, N-acetyl-DL-glutamic acid (*p* < 0.001) and N-acetyl-glutamine (*p* < 0.001) had significantly higher AUC for DI, whereas glutamine (*p* < 0.001) and SDMA (*p* < 0.001) had significantly lower AUC. Comparing HI versus LO, ornithine (*p* < 0.05), creatine (*p* < 0.001) and citrulline (*p* < 0.05) had significantly lower AUC during HI. For the δAUC differences, creatine and ornithine had significantly lower δAUC in both DI compared with BR (both *p* < 0.01) and HI compared with LO (both *p* < 0.05). 

Additionally, ratios of key metabolites in the Arginine-NO pathway also showed significant differences (Table 3): glutamine-to-glutamate ratio had significantly lower AUC in DI than BR (*p* < 0.001); arginine-to-ADMA ratio had significantly higher AUC in DI than BR (*p* < 0.001), etc. Furthermore, there were other metabolites that also showed notably significant differences: examples can be seen as N6,N6,N6-trimethyllysine (TML), which had significantly higher AUC in DI than BR (*p* < 0.001), and lysine, which showed significantly lower AUC in DI than BR (*p* < 0.001).

## 4. Discussion

While we have previously reported the interaction of low or high GI meals with the timing of food intake on markers of glucose homeostasis [14], in this secondary analysis, we have additionally investigated targeted metabolomics to identify metabolites and pathways which may be linked to differences in glucose homeostasis observed between various conditions. Indeed, we have found that 29 out of 234 (approx. 12%) of these plasma metabolites had significant differences in postprandial AUC between BR and DI groups. A previous study by Sato et al. (2018) which investigated the interactions between intake timing (morning versus evening) and diet (high-carbohydrate versus high-fat), also derived a clear separation of serum metabolites between morning and evening [12]. The smaller proportion of metabolites which are different between dinner and breakfast in our study may be related to the semi-targeted nature of our analyses. Notably, in our study, the difference between breakfast and dinner was far more pronounced than the comparisons between low and high GI meals, as shown in the PLS-DA plot and will be described in the subsequent paragraph. It was also noted that the differences in postprandial metabolite levels due to variations in meal GI were relatively minimal and largely non-significant, with only 5 out of 234 (2%) metabolites exhibiting significant differences between the HI and LO groups, thus suggesting that GI per se had comparatively lower effects on our measured metabolites. Additionally, all of these five metabolites exhibited significantly lower postprandial concentrations following the high-GI meal.

Among the various pathways considered, the arginine-NO pathway had the greatest abundance of metabolites, which showed significant differences based on the time of the day (i.e., morning or evening), implying that this pathway is closely governed by peripheral circadian rhythms and that the regulation of NO balance may be more susceptible to the impacts of late eating. The accumulation of nitrosative stress is increasingly recognised as an underlying mechanism contributing to heart failure and cardiac dysfunction [18,19], emphasising that late eating per se can potentially increase nitrosative stress leading to increased cardiovascular disease risk. Additionally, arginine also exerts strong insulinotropic effects in the presence of glucose by acting directly on pancreatic beta cells, highlighting its potential role in the circadian control of glycemic response and insulin action [20]. 

We have reported higher levels of plasma SDMA and a lower arginine-to-ADMA ratio following breakfast as compared to dinner. These findings are corroborated with other metabolomics studies, which reported significantly higher SDMA levels at baseline in the morning as compared to the evening, supporting that its diurnal variability is largely distinct [21,22]. The arginine-to-ADMA ratio has been reported as a more representative indicator of NO bioavailability as compared to either arginine or ADMA alone [23]. As an antagonist of the NO-mediated vasodilation [24], the diurnal variability of ADMA in relation to arginine within normal ranges may play a potential role in the circadian regulation of blood pressure, which peaks during the mid-morning and progressively lowers from the late afternoon to night-time in normotensive individuals [25].

While a higher arginine-to-ADMA ratio in the evening may be integral for NO-mediated circadian vasodilation, this, in turn, presents a milieu of increased susceptibility to oxidative damage driven by mistimed food intake. As evening meal intake, in addition, may contribute to a greater extent of postprandial inflammation as compared to morning intake [26], this could potentially increase the expression of inducible nitric oxide synthase (iNOS), leading up to an abnormal increase in NO production from arginine at night [27]. Exposure to higher levels of ROS as a signal response for the insulin action [28] further contributes to insulin resistance, vascular damage and cardiometabolic risk.

We have also reported significantly lower glutamine and higher N-acetyl-glutamine levels in the DI period as compared to BR, suggesting increased glutamine acetylation in the evening. Glutamine has been reported to be negatively correlated to type 2 diabetes incidence [29] and improved beta-cell function in people with type 2 diabetes [30]. Conversely, low glutamine levels are also an indication of an increased catabolic stress [31]. This, together with the lower glutamine-to-glutamate ratio during dinner (*p* < 0.001) in this study, indicates a compromised circadian-related metabolic state during the evening [32]. The glutamine-to-glutamate ratio has also been reported to be inversely associated with HbA1c levels and incident type 2 diabetes, while another study found lower 24-h glutamine-to-glutamate ratio in patients with type 2 diabetes as compared with lean volunteers, further supporting the link between late meal intake with poorer glucose homeostasis and type 2 diabetes risk [22,33,34]. 

While baseline creatine levels were relatively higher during the evening, there was a significantly greater reduction in postprandial plasma creatine following dinner intake as compared to breakfast intake. The HI group also produced a significantly greater decrease in creatine levels as compared to the LO group, amounting to a significantly lower AUC following a high-GI meal. Taken together, this may suggest that exposure to high-GI meal intake, as well as dinner consumption, leads to greater overall creatine utilisation, potentially to counter hyperglycemia-induced oxidative stress [35].

In addition, plasma levels of amino acids, such as ornithine and citrulline, were also shown to be significantly lower following a high-GI meal as compared to low-GI, irrespective of intake timing. A reduction in plasma citrulline may allude to increased nitrosative stress, consequently increasing the risk of insulin resistance and/or cardiovascular disease in the longer term [36,37]. Ornithine, the precursor of citrulline in the urea cycle, was also reduced to a significantly greater extent following the high-GI meal (as compared to the low-GI meal) and dinner timing (in comparison with breakfast). The reduced ornithine levels observed in our study may potentially be associated with postprandial inflammation [37], induced by heightened glycemia following a high-GI meal and dinner intake, although postprandial interleukin-6 (IL-6) was not different between the four treatments (data not shown). Perturbations in amino acid metabolism, as suggested by abnormal elevations or reductions in plasma amino acids, are also associated with the development of insulin resistance and type 2 diabetes [36,37,38]. Highlighting their potential role as biomarkers or therapeutic targets in the prevention or management of chronic metabolic conditions.

We have also identified other metabolites outside of the arginine-NO pathway with prominent changes in plasma AUC levels between breakfast and dinner. TML is a pro-inflammatory precursor of trimethylamine N-oxide (TMAO), with multiple reports of its strong associations with cardiovascular events and coronary heart disease [39,40,41]. Its precursor amino acid lysine on, the other hand, has been reported to be inversely associated with early metabolic syndrome and inflammatory markers, such as IL-6 and oxidised LDL, as well as GI [42]. Lower lysine levels were also associated with higher glucose levels in the type 2 diabetes serum metabotype, relative to those with normal glucose tolerance and impaired glucose regulation [43]. We have observed significantly higher TML concomitant with lower lysine levels, irrespective of GI, during dinner—suggesting a compromised metabolic state during dinner compared with breakfast. Lysine has also been reported to be insulinotropic and positively correlated with glucagon-like peptide-1 (GLP-1) in patients with type 2 diabetes, adding to the explicably lower breakfast glycemic excursions through greater precursor and insulin signaling [30].

Other human metabolomics studies have also reported wide variations in the fasting plasma metabolome between normoglycemic individuals, people with metabolic syndrome, and patients with diabetes, emphasizing the link between disturbances in the regular variability of plasma metabolites and impaired glucose metabolism, as well as cardiometabolic disease risk [36,37,38,44]. Nevertheless, these observations may also be an outcome of reverse causality—such as an abnormal increase in secondary metabolites from pathways, as an effect of increased glucose flux arising from hyperglycemia [38].

### 4.1. Novelty

To our knowledge, this is the first 2 × 2 crossover study assessing the interactions of both GI and timing of intake in the assessment of targeted metabolomics. While majority of metabolomics studies involve the collection of blood samples in the morning after an overnight fast, the analysis of blood samples taken during the evening in our study enabled the assessment of metabolite fluctuations throughout distinct periods either at the beginning or at the end of the day. The differences in metabolites during breakfast and dinner (morning/evening) further points to the importance of measuring physiological and biochemical states not only during the fasted state in the morning, but also during the evening to assess metabolic state, their relation to dietary exposure and their subsequent associations with health or disease risk.

The strengths of our study include the use of a crossover design, which accounts for intra-individual variabilities in metabolomic expression whereby all volunteers completed all four sessions. Moreover, the use of a mixed meal in the assessment of postprandial metabolomics is more representative of postprandial metabolism based on a real life dietary setting, in contrast to simpler, conventional tests, such as an oral glucose tolerance test (OGTT) [45]. All TMs were also provided by the study team, including identical pre-meals that were served prior to consumption of the TMs (i.e., SM dinner provided before the TM breakfast; SM lunch provided before the TM dinner), thereby facilitating the collection of samples under strictly controlled conditions.

Nevertheless, we have considered certain limitations. The targeted metabolomics analysed in our study are restricted only to certain groups of metabolites. As such, expanding the range of metabolites analysed would have provided greater insights to the different aspects of cardiometabolic health affected by high or low GI, and/or intake timing. Additionally, as our study only takes in account the acute changes in metabolite levels, the longer-term consequences of these variations remain to be determined. Furthermore, only the pertinent changes in certain metabolites and not all significantly different metabolites have been discussed here. Nonetheless, we have presented the postprandial plots for all metabolites with significant *p*-values in our study (Supplementary Figure S1) for future reference, which may enable the identification of molecular and metabolomic signatures associated with physiological changes in relation to differences in intake timing and/or variations in meal GI. Participant chronotype information could also have been assessed to minimise any potential confounding, as those with late chronotypes may be predisposed to greater circadian misalignment and cardiometabolic disease risk, as well as a less favourable metabolic response towards breakfast consumption relative to early chronotypes [21,46].

### 4.2. Future Directions

Our study indicated that in general, the metabolic state of individuals points towards greater cardiometabolic disease risk during the evening, as compared to the morning period. Under this milieu, there is a potential opportunity for the introduction of chronobiotic or chronopharmaceutical supplements, in order to counterbalance the metabolic perturbations that are associated with circadian changes during the later part of the day. Indeed, there are evidence from some animal and human studies which have indicated greater benefits of consuming non-nutrient bioactive compounds at resting phase (night-time for humans) as compared with the active phase [47,48,49]. Hence, there may be a rationale for consuming relatively greater proportions of nutrient-dense foods at night, potentially in mitigating cardiometabolic risk. Postprandial metabolomics (untargeted) may also be further applied in future chrononutrition studies to investigate the circadian interactions that occur following the consumption of other macronutrients and micronutrients. 

## Figures and Tables

**Table 1:** Differences in AUC and δAUC between dinner and breakfast.

Metabolite	Effect Size ^1^	*p* ^2^	Relevant Metabolic Pathway
AUC			
N6,N6,N6-trimethyllysine (feature 1)	1.70	7.08 × 10^−27^ ***	Lysine metabolism, carnitine synthesis
Trigonelline	1.44	1.58 × 10^−13^ ***	Tryptophan-nicotinamide metabolism
N-acetyl-DL-glutamic acid	1.38	1.09 × 10^−12^ ***	Arginine-NO pathway
N-acetyl-glutamine (feature 1)	1.36	2.34 × 10^−12^ ***	Arginine-NO pathway
Hydroxypyruvate/3-hydroxybutyrate	1.09	2.43 × 10^−6^ ***	Serine metabolism/fatty acid biosynthesis, ketone metabolism
Pyrrolidinone	0.96	4.48 × 10^−5^ ***	-
Imidazole	0.94	4.25 × 10^−5^ ***	Histidine catabolism
Glycerophosphocholine	0.87	1.06 × 10^−4^ ***	Retinol and choline metabolism
Pyrazole	0.80	1.06 × 10^−4^ ***	-
Caffeine	0.76	9.82 × 10^−4^ ***	Tryptophan-nicotinamide metabolism
Acetylglycine/Guanidineacetate	0.67	5.25 × 10^−3^ **	Guanidineacetate: arginine, proline, glycine, threonine and serine metabolism
Linoleate (feature 1)	0.63	0.03 *	-
Acetylputrescine (feature 1)	0.60	0.04 *	Arginine/proline metabolism
Pipecolic acid (feature 2)	0.58	0.04 *	Lysine metabolism (by intestinal microflora)
(S)-malate/Glutaric acid	0.51	0.01 *	Glucose metabolism/Glutamate, tryptophan and lysine metabolism
Ornithine	0.47	0.08	Arginine-NO pathway, urea cycle
1-Methyladenosine	−0.51	0.10	-
N6-acetyllysine	−0.54	0.02 *	Lysine metabolism
N-acetyl-glutamine (feature 2)	−0.56	0.02 *	Arginine-NO pathway
ADMA	−0.61	0.06	Arginine-NO pathway
Histamine	−0.62	0.03 *	Histidine catabolism
Histidine	−0.70	0.02 *	Histidine metabolism
Cytosine (feature 1)	−0.75	0.01 *	Pyrimidine synthesis/salvage pathway
Serine	−0.79	6.51 × 10^−3^ **	Purine and pyrimidine synthesis pathway
Homocysteine (feature 2)	−0.82	1.44 × 10^−3^ **	Methionine catabolism
SDMA	−0.99	1.02 × 10^−4^ ***	Arginine-NO pathway
Oxypurinol	−1.00	1.82 × 10^−5^ ***	Purine synthesis/salvage pathway
Glutamine	−1.09	1.82 × 10^−5^ ***	Arginine-NO pathway
Methylnicotinamide	−1.24	3.32 × 10^−11^ ***	Tryptophan-nicotinamide metabolism
Lysine	−1.28	7.73 × 10^−10^ ***	Lysine metabolism, carnitine synthesis
Uracil	−1.47	3.32 × 10^−11^ ***	Pyrimidine synthesis/salvage pathway
Uridine	−1.48	1.12 × 10^−11^ ***	Pyrimidine synthesis/salvage pathway
δAUC ^3^			
Uridine	0.73	0.08	Pyrimidine synthesis/salvage pathway
Methylnicotinamide	−0.71	0.08	Tryptophan-nicotinamide metabolism
Lysine	−0.72	0.08	Lysine metabolism, carnitine synthesis
Creatine	−0.93	1.72 × 10^−3^ **	Arginine-NO pathway
Ornithine	−1.12	1.52 × 10^−5^ ***	Arginine-NO pathway, urea cycle

^1^: Effect size is the estimated coefficient of Time term in LMM and GLM, which refers to (δ)AUC(DI)–(δ)AUC(BR). ^2^: ***: *p* < 0.001; **: 0.001 < *p* < 0.01; *: 0.01 < *p* < 0.05. ^3^: Uridine level showed an increase after dinner and a minor reduction after breakfast; Methylnicotinamide level showed a greater increase after breakfast; Lysine, Creatine, and Ornithine levels showed greater reduction after dinner (Supplementary Figure S1). AUC: area under the curve; (δ)AUC: baseline-corrected area under the curve; NO: nitric oxide; ADMA: asymmetric dimethylarginine; SDMA: symmetric dimethylarginine.

**Table 2:** Differences in AUC and δAUC between high and low-GI.

Metabolite	Effect Size ^1^	*p* ^2^	Relevant Metabolic Pathway
AUC			
Lysine	−0.51	0.10	Lysine metabolism
Imidazole	−0.60	0.06	Histidine catabolism
Trans-4-hydroxyproline (feature 1)	−0.65	0.09	Collagen synthesis/degradation, cell signalling
Arginine	−0.66	0.06	Arginine-NO pathway
Ornithine	−0.68	0.02 *	Arginine-NO pathway, urea cycle
Citrulline	−0.69	0.04 *	Arginine-NO pathway, urea cycle
Histamine	−0.74	0.03 *	Histidine catabolism
Metanephrine	−0.95	5.81 × 10^−5^ ***	Tyrosine metabolism
Creatine	−1.15	5.05 × 10^−6^ ***	Creatine metabolism
δAUC ^3^			
Creatine	−0.93	3.78 × 10^−3^ **	Arginine-NO pathway
Ornithine	−0.77	0.02 *	Arginine-NO pathway, urea cycle

^1^: Effect size is the estimated coefficient of GI term in LMM and GLM, which refers to (δ)AUC_(HI)_–(δ)AUC_(LO)_. ^2^: ***: *p* < 0.001; **: 0.001 < *p* < 0.01; *: 0.01 < *p* < 0.05. ^3^: Creatine and Ornithine levels showed greater reduction after a high-GI meal (Supplementary Figure S1). AUC: area under the curve; (δ)AUC: baseline-corrected area under the curve; NO: nitric oxide.

**Table 3:** Key metabolite ratios in the arginine-NO pathway with significant differences between dinner and breakfast.

Metabolite Ratio ^1^	Effect Size ^2^	*p* ^3^
Arginine:SDMA	1.23	1.91 × 10^−10^ ***
Arginine:ADMA	0.88	2.19 × 10^−4^ ***
Arginine:NG,NG-Dimethylarginine	0.66	0.01 *
Arginine:Dimethylarginine	0.58	0.04 *
Homoarginine:Lysine	0.50	4.70 × 10^−2^ *
Glutamine:Glutamate	−0.93	7.61 × 10^−5^ ***
Glutamate:Acetylglutamine	−1.37	8.35 × 10^−13^ ***
Lysine:Arginine	−1.42	5.45 × 10^−19^ ***
Glutamate:Acetylglutamate	−1.47	2.37 × 10^−14^ ***
Glutamine:Acetylglutamate	−1.76	6.88 × 10^−26^ ***
Glutamine:Acetylglutamine	−1.77	3.11 × 10^−27^ ***

^1^: Metabolite ratio is shown as metabolite 1:metabolite 2, which means metabolite 1-to-metabolite 2 ratios. ^2^: Effect size is the estimated coefficient of *Time* term in LMM, which refers to AUC_(DI)_–AUC_(BR)_. ^3^: ***: *p* < 0.001; *: 0.01 < *p* < 0.05. AUC: area under the curve; (δ)AUC: baseline-corrected area under the curve; NO: nitric oxide; ADMA: asymmetric dimethylarginine; SDMA: symmetric dimethylarginine.

## Data Availability

De-identified participant data are available on reasonable request, within the regulatory framework of the authors’ institutional policies, by emailing the corresponding authors at ‘sumanto_haldar@sifbi.a-star.edu.sg’ and ‘mdccld@nus.edu.sg’.

## Supplementary Materials

The following supporting information can be downloaded at: https://www.mdpi.com/article/10.3390/metabo13040490/s1. Methods: Metabolite peak integration; Column-specific batch correction; Quality control by CoV and D-ratio. Table S1: SRM Table of analytes and Internal standard (ISTD). Table S2: Detailed composition of the test meals (TM) and standardized meals (SM). Figure S1 (a). *p*-value distribution of average intensity comparison for metabolites measured in 2 columns. (b). Distribution of metabolites’ CoV and D-ratio before and after batch correction. Figure S2: Level-timeline plots of the metabolites with significant *p*-values. Figure S3: Simple pathway map of Arginine-NO pathway.
