# Supplementary material for: Chronometabolism: The Timing of the Consumption of Meals Has a Greater Influence Than Glycemic Index (GI) on the Postprandial Metabolome
Source: Metabolites. 2023 Mar 29;13(4):490. doi: 10.3390/metabo13040490 (PMC10143625; doi:10.3390/metabo13040490)
Supplement: Supplementary file 1 [file metabolites-13-00490-s001.zip › metabolites-2288437-supplementary.pdf]

Supplementary Methods

Metabolite peak integration

During peak integration, all analytes were normalized to the peak height of their mapping ISTD. To perform this, in MRMKit parameter setting, "ISTD_trace_all" was set to 1, "normalize" was set to 1 [1]. MRMKit would then scale the analyte peak to the corresponding ISTD peak, the intensity output of each analyte was calculated as below:

$$Analyte\ Intensity = \frac{Analyte\ peak\ height}{ISTD\ peak\ height} \tag{1}$$

The intensity values would then be included in the initial data for down-stream statistical analysis.

Column-specific batch correction

Our assay was performed by using 2 columns with column-switching mode, therefore column-specific batch effect would exist in addition to common batch effect. We hereby applied column-specific batch correction, during which each column would be treated as a separate batch. There would be different initial batches for different runs, while within each initial batch/run, there would also be 2 different batches because they were measured by 2 columns. Finally, the number of batches input to MRMKit for processing would be double the number of actual runs. Then with "batch_correction" set to 1 in MRMKit, the column-specific batch correction would be performed.

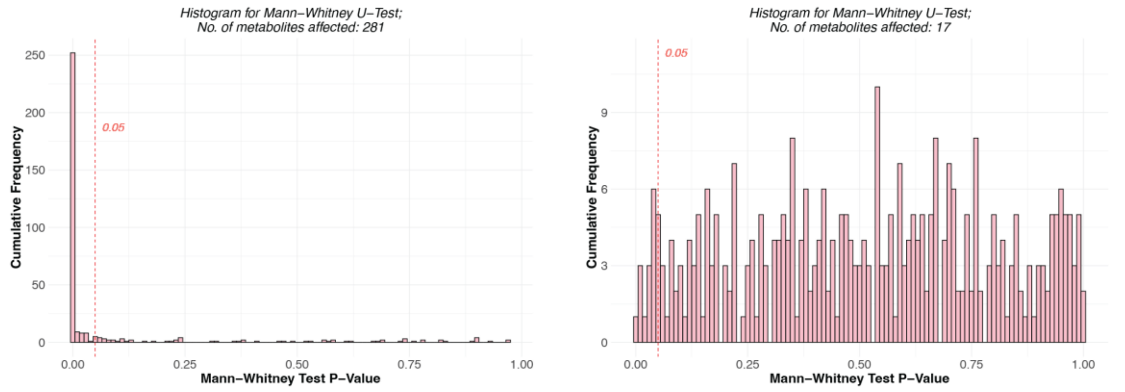

Before batch correction   After batch correction

(a). *p*-value distribution of average intensity comparison for metabolites measured in 2 columns (left: before batch correction; right: after batch).

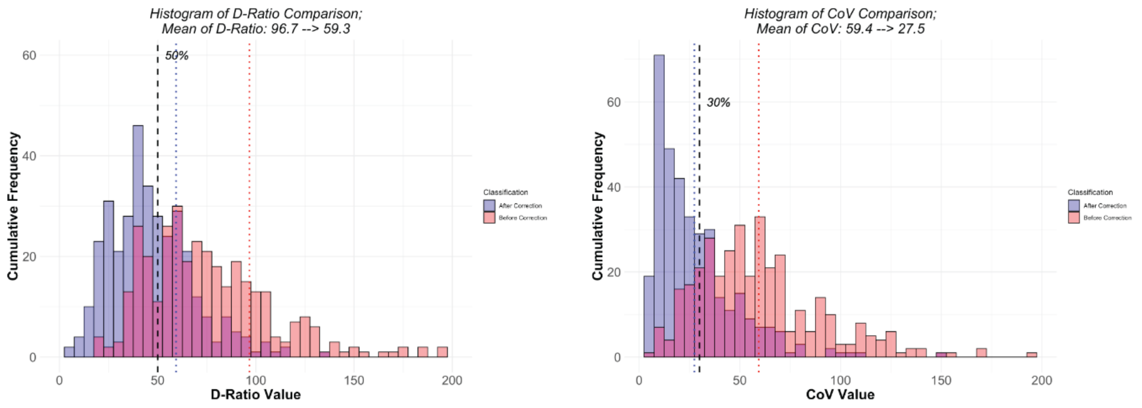

(b). Distribution of metabolites' CoV and D-ratio before and after batch correction (left: D-ratio distribution; right: CoV distribution).

Figure S1 (a). *p*-value distribution of average intensity comparison for metabolites measured in 2 columns (left: before batch correction; right: after batch). **(b)**. Distribution of metabolites' CoV and D-ratio before and after batch correction (left: D-ratio distribution; right: CoV distribution).

To check whether this batch correction method improved data quality, we observed the average intensity comparison result of same analytes measured by the 2 columns before and after batch correction. As shown in Figure S1 **(a)**, before batch correction, most of the p-values were less than 0.05, indicating that there're significant difference between the 2 columns. While after the correction, the p-values were more evenly distributed, showing that this column-specific batch correction did decrease the batch effect caused by using different columns. Analytes' CoV and D-ratio also improved after correction (Figure S1 **(b)**).

Quality control by CoV and D-ratio

Data quality control was performed by (1) calculating coefficient of variation (CoV), dispersion ratio (D-ratio) [2] (2) filtering out low-quality data based on CoV and D-ratio threshold. For each indicated batch, CoV was calculated as:

$$CoV = \frac{Standard\ Deviation\ (SD)\ across\ all\ pooled\ samples}{Mean\ across\ all\ pooled\ samples} \quad (2)$$

which indicates the internal variance of repeated measurements.

D-ratio was calculated as:

$$D-ratio = \frac{Standard\ Deviation\ (SD)\ across\ all\ pooled\ samples}{Standard\ Deviation\ (SD)\ across\ all\ subjects\ samples} = \frac{Non-biological\ variance}{Biological\ variance} \quad (3)$$

which shows the departure of non-biological variance from biological variance. Hence, analytes with smaller CoV and D-ratio were expected to have better and more reliable data quality. In our assay, analytes with CoV > 30% and D-Ratio > 50% were removed.

Table S1. SRM Table of analytes and Internal standard (ISTD)

Name	Precursor Ion (m/z)	Product Ion (m/z)	Ret. Time (s)	Collision Energy (V)	Cell Acc. Voltage (V)	Polarity	Remark
			**Analyte**				
(S)-Malate/Glutaric acid	133.1	115.1	360	10	5	Pos	
1-Methyladenosine	281.8	150	196.5	27	5	Pos	
1-Methylhistidine	170.1	124.1	321.1	14	5	Pos	
11-Deoxycortisol	347.3	97.1	312	30	5	Pos	
12-Deoxycortisol	347.4	311.3	46	13	5	Pos	Removed after QC
17alpha-Hydroxyprogesterone	331.2	109.1	46	30	5	Pos	
2-Amino-2-methylpropanoate	104.1	45.1	135.6	10	5	Pos	
2-Aminobutyrate/Dimethylglycine	104.1	58.1	134.4	10	5	Pos	
2-Aminoisbutyric acid	104.1	58.1	134.4	10	5	Pos	
2-Aminooctanoic acid	160	55.3	159.8	21	5	Pos	Removed after QC
2-Aminopimelic acid/4-Aminopimelic acid	174.1	130	207	12	5	Neg	Removed after manual review
2-Hydrobenzoic acid/4- Hydrobenzoic acid	137	93.2	72.6	12	5	Neg	
2-hydroxyisobutyric acid	105.1	77	168	10	5	Pos	
2-Oxoglutarate/Phenylpropiolic acid	145	101	269.6	13	5	Neg	
2,3-dihydroxybenzoic acid	153	109	120	19	5	Neg	
3-Amino-2-methylpropanoic acid (BAIBA)	103.9	86	210	3	5	Pos	
3-Aminoisobutanoate	104.2	86.1	145.8	10	5	Pos	
3-Carboxy-4-methyl-5-propyl-2-furanpropanoic acid (CMPF) (feature 1)	239	195	52.1	10	5	Neg	
3-Carboxy-4-methyl-5-propyl-2-furanpropanoic acid (CMPF) (feature 2)	239	195	65	10	5	Neg	Removed after QC
3-Chlorotyrosine	216.1	170	175.8	12	5	Pos	
3-Hydroxybutyrate/Hydroxypyruvate	103	59	72	5	5	Neg	
3-Hydroxyisovalerylcarnitine	262.1	84.9	160.8	22	5	Pos	

Table S1. Continued.

3-Indolepropionate	189.9	129.9	48.6	20	5	Pos	Removed after QC
3-Methoxytyramine	303.1	151.1	44.4	25	5	Pos	Removed after QC
3-Methylhistamine	126.2	96.1	342	21	5	Pos	
3-Methylhistidine	170.1	126	348.6	12	5	Pos	
3-Nitrotyrosine (feature 1)	227.1	168.1	150	16	5	Pos	
3-Nitrotyrosine (feature 2)	227.1	181.8	156	16	5	Pos	
3-Nitrotyrosine (feature 3)	227.1	180.8	216	12	5	Neg	
3-Phosphoserine	186	88	87	15	5	Pos	Removed after QC
4-Aminobutyric Acid	104.01	69	198	22	5	Pos	Removed after QC
4-Guanidinobutyric acid	146.1	86	20.6	25	5	Pos	
4-Hydroxy-L-Proline	130.1	87.9	207.2	10	5	Neg	
4-Pyridoxic acid//Epinephrine	182.4	138.1	108	10	5	Neg	
5-Aminopentanoate	118.2	55.1	207.2	16	5	Pos	
5-Hydroxyindoleacetate	192.1	146	169.3	15	5	Pos	
5-Hydroxytryptophan/Serotonin	177.1	160.1	274.8	20	5	Pos	
5-Methylcytosine hydrochloride	126.2	109	354.9	20	5	Pos	
6-Aminouracil	127.9	67.8	201.6	20	5	Pos	
6'-Hydroxynicotinate	140.1	94	213	22	5	Pos	Removed after manual review
7-Methylguanosine	298	166	163.2	24	5	Pos	
7alpha-Hydroxy-4-cholesten-3-one (7-HCO)	401.5	177	45	24	5	Pos	Removed after manual review
7alpha,12alpha-Dihydroxycholest-4-en-3-one (7,12-DHCO)	417.1	381.2	47.3	20	5	Pos	
8-Hydroxydeoxyguanosine	284.3	168.1	4	10	5	Pos	Removed after QC
8-Isoprostane	353.4	193.2	54	30	5	Neg	
Abscisic acid	265.1	247.1	212.9	10	5	Pos	
Acadesine	259	110	153	24	5	Pos	Removed after QC

Table S1. Continued.

Acetaminophen	152	110	228.6	20	5	Pos	
Acetylcarnitine (C2)	260	85.1	76.7	26	5	Pos	Removed after manual review
Acetylcholine	146.1	87	189.7	14	5	Pos	
Acetylglycine/Guanidineacetate	118	76	263.6	10	5	Pos	
Acetylphosphate	139	79	168	24	5	Neg	
Acetylputrescine (feature 1)	131.1	114.1	168	10	5	Pos	detected RT:150
Acetylputrescine (feature 2)	131.1	114.1	168	10	5	Pos	detected RT:197
Aconitate	173	85	228	10	5	Neg	
Adenine	136	92	209.3	30	5	Pos	
Adenosine	268	136	166.2	19	5	Pos	Removed after QC
Adipoylcarnitine (C6DC)	290	85.1	47	35	5	Pos	
ADMA	203.2	46.2	294.9	17	5	Pos	
Agmatine	131	72	210	15	5	Pos	
Alanine	90.1	44	257.5	6	5	Pos	
Allantoic acid	177	61	360	10	5	Pos	
Allantoin (feature1)	157	96.9	164.6	10	3	Neg	
Allantoin (feature2)	157	114	166	8	3	Neg	
Allantoin (feature3)	159.1	116	137.6	5	5	Pos	Removed after QC
Alpha-Aminoadipate	162.2	127	213	20	5	Pos	
Alpha-Tocopherol (Vitamin E)	431.4	165	37	17	5	Pos	
Aminoisobutyrate	104.1	86	149.4	16	5	Pos	
Androstenedione	287.1	97.1	60	22	5	Pos	
Arachidic carnitine (C20:0)	456.7	85	53.4	25	5	Pos	
Arginine	175	70	363.6	25	5	Pos	
Argininosuccinate	289.1	271.1	209.8	10	5	Neg	
Asparagine	133	74	267.6	13	5	Pos	
Behenic carnitine (C22:0)	484.7	85	40	25	5	Pos	
Betaine	118	58	190	25	5	Pos	

Table S1. Continued.

Betaine aldehyde	102	58	218.4	21	5	Pos	Removed after QC
Bilirubin	583.3	285	40.8	36	5	Neg	
Biotin	243	42	171	55	5	Neg	Removed after QC
Butyrylcarnitine	232.1	84.9	105	20	5	Pos	
Caffeine	195.2	138.1	49.8	20	5	Pos	
CDP-ethanolamine	445	273	41.4	29	5	Neg	Removed after QC
Cervonyl carnitine (C22:6)	472.7	85	114	25	5	Pos	
Cholesteryl sulfate	465.2	97	54.4	39	5	Neg	Removed after QC
Cholic acid (CA)	407.2	345.2	42.9	32	5	Neg	
Choline	104	60	150	17	5	Pos	
Cis-5-tietradecenoylcarnitine	370.3	85	66	15	5	Pos	
Citramalate	149.1	93.1	36.7	18	5	Pos	
Citrulline	176.1	70.1	370.8	15	5	Pos	
Corticosterone	347.2	121.2	47.4	22	5	Pos	Removed after QC
Cortisone	361.2	163	48	22	5	Pos	Removed after manual review
Creatine (feature 1)	132.1	44.3	176.4	21	5	Pos	detected RT: 174
Creatine (feature 2)	132.1	44.3	176.4	21	5	Pos	detected RT: 183; Removed after manual review
Creatinine	114	44.3	151	19	5	Pos	Removed after manual review
Cyanocobalamin	678.3	147	97.8	32	5	Pos	Removed after QC
Cys-Gly, oxidized	355.1	176.8	37.2	20	5	Pos	Removed after QC
Cystathionine	221.1	120.1	132	12	5	Pos	
Cysteine	122.1	76.1	295.8	13	5	Pos	
Cysteine-sulfinic acid	154.1	136	251.8	5	5	Pos	
Cystine	241.2	151.9	338.5	9	7	Pos	
Cytosine (feature 1)	112	95	138	20	5	Pos	
Cytosine (feature 2)	112	95	155	20	5	Pos	Removed after QC
D-Erythrose-4-phosphate	199	97	193.3	19	5	Neg	Removed after manual review

Table S1. Continued.

D-Glucosamine/S-carboxymethyl-L-cysteine	180.1	163	158.4	10	5	Pos	Removed after QC
D-Glyceraldehyde 3-phosphate	169.1	97	210.6	14	5	Neg	
D-Glyceric acid	107	79	166.8	10	5	Pos	
D-Pantothenic acid	220.2	90.1	92.4	10	5	Pos	
Decenoylcarnitine	314.5	85	60	24	5	Pos	
Deoxyadenosine	252	136	150.6	22	5	Pos	
Deoxycarnitine	146.2	87	183	16	5	Pos	Removed after manual review
Deoxycholic acid (DCA)	391.2	345.2	48.6	36	5	Neg	Removed after QC
Dihydroorotate	157	113	210	14	5	Neg	
Dihydroxyacetone phosphate	183.2	165.1	39	10	5	Pos	
Dimethylarginine	203.2	70.1	293.4	20	5	Pos	
Dityrosine	361.3	315.1	49.8	25	5	Pos	
Dodecenoylcarnitine (C12:1)	342.5	85	58.8	25	5	Pos	Removed after manual review
Dopamine	154.2	91.2	94.8	22	5	Pos	Removed after QC
dUMP	307	195	52	18	5	Neg	Removed after QC
Epinephrine	184.1	166	69	11	5	Pos	
Ergothioneine	230	127	246	20	5	Pos	
Ethyl-3-ureidopropinoate	161.1	120	165.3	10	5	Pos	
Flavone	223	121	52.2	29	5	Pos	
Free carnitine (C0)	162	103	217.8	22	5	Pos	
Fumarate/Maleic acid	115	71	360	13	5	Neg	Removed after QC
Galactitol	183.1	165.1	204	10	5	Pos	Removed after QC
Gluconolactone	177	99	225	10	5	Neg	
Glutaconic acid	129	85	163.6	11	5	Neg	
Glutamic acid	148.1	84.1	282	17	5	Pos	
Glutamine	147.1	84.1	360	17	5	Pos	
Glutarylcarnitine (C5DC)/Glutarylcarnitine (C6-OH)	276	85.1	201.8	40	5	Pos	

Table S1. Continued.

Glutathione-oxidized (GSSG)	613.2	355.2	37	22	5	Pos	
Glutathione-reduced (GSH)	308.1	84	43.2	18	5	Pos	Removed after QC
Glycerophosphocholine	258.1	104	270	16	5	Pos	
Glycine	76.4	58.1	192	10	5	Pos	
Glycochenodeoxycholic acid (GCDCA)/Glycodeoxycholic acid (GDCA)/Glycoursodeoxycholic acid (GUDCA)	448.3	74	85.6	10	5	Neg	
Glycocholic acid (GCA)	464.4	74.2	111	30	5	Neg	
Glycolaldehyde dimer	121.1	77.1	166.8	21	5	Pos	
Glycolate/Propylene glycol (feature 1)	77.1	59	208.1	10	5	Pos	
Glycolate/Propylene glycol (feature 2)	77.2	59.1	207.4	10	5	Pos	
Glyoxylate	73	45	198	8	5	Pos	
Guanosine	284.1	151.9	197.9	23	5	Pos	
Heptanoyl-l-carnitine (C7:0)	274.3	85	76	20	5	Pos	
Hercynine	198	154.1	292.8	9	4	Pos	
Hexadecenoyl carnitine (C16:1)	398.3	85	66	15	5	Pos	
Hexenoylcarnitine (C6:1)/Hexenoylcarnitine (C6)	258	85.1	96	35	5	Pos	
Hippurate	178	77	120	20	5	Neg	Removed after manual review
Histamine	112.1	95	360	15	5	Pos	
Histidine	156.1	110.1	367.2	14	5	Pos	
Homoarginine (hArg) (feature 1)	189.2	144	321.6	15	5	Pos	
Homoarginine (hArg) (feature 2)	189.2	144	351	15	5	Pos	Removed after manual review
Homocysteine (feature 1)	136.1	91	108	19	5	Pos	Removed after QC
Homocysteine (feature 2)	136.1	91	162	19	5	Pos	
Homocysteine (feature 3)	136.1	90.2	204	19	5	Pos	
Hydroxybutyryl-carnitine (C4-OH)	248	85.1	177.3	30	5	Pos	
Hydroxydodecenoylcarnitine	358.5	85	60.3	25	5	Pos	Removed after manual review
Hydroxyhexadeceneoylcarnitine	414.3	85	29.1	25	5	Pos	

Table S1. Continued.

Hydroxyproline	132	68.2	252	19	5	Pos	Removed after QC
Hypotaurine	110.1	82.1	360	12	5	Pos	
Hypoxanthine	134.9	91.9	125.4	18	5	Neg	
Imidazole	69	41.4	184.8	10	5	Pos	
Indole	118	91	177.3	26	5	Pos	
Isovaleryl-carnitine (3-M-C4:0)	246.1	85	92.9	15	5	Pos	
Kynurenate	190.1	144.1	345	15	5	Pos	
Kynurenine	209.2	192	167.8	10	5	Pos	
L-Aspartic Acid	134	74	212.1	12	5	Pos	
L-DOPA	198.1	152.1	48	8	5	Pos	Removed after QC
L-homocysteine thiolactone	118.2	100	128.9	14	5	Pos	Removed after QC
L-Ornithine	133.1	70.1	374.2	16	5	Pos	
Lactate	89	43.2	100.9	16	5	Neg	
Lauroylcarnitine	344.2	285	72.7	16	5	Pos	Removed after manual review
Leucine/Isoleucine (feature 1)	132.1	86	183	13	5	Pos	detected RT: 174
Leucine/Isoleucine (feature 2)	132.1	86	183	13	5	Pos	detected RT: 183
Linoleate (feature 1)	279.2	279.2	41.4	2	5	Neg	
Linoleate (feature 2)	279.2	279.2	69	2	5	Neg	
Lithocholic acid (LCA)	498.3	80	127.4	70	5	Neg	
LPC 20:0	552.4	184	103.6	25	5	Pos	Removed after QC
LPC 20:1	550.4	184	104.8	25	5	Pos	
LPC 20:3	546.4	184	106.1	25	5	Pos	Removed after manual review
LPC 20:4	544.3	184	106.1	25	5	Pos	Removed after QC
LPC 20:5	542.3	184	109.8	25	5	Pos	
LPC 22:2	576.4	184	102	25	5	Pos	Removed after QC
LPC 22:6	568.3	184	104.8	25	5	Pos	Removed after manual review
LPC 24:0	608.5	184	98.5	25	5	Pos	Removed after QC
LPC 26:0	636.5	184	90	25	5	Pos	Removed after QC

Table S1. Continued.

LPC 28:1	662.5	184	72	25	5	Pos	Removed after QC
Lysine	147	84	361.8	14	5	Pos	
Maleic acid	115	71	120	13	5	Neg	
Malonylcarnitine (C3-DC)	248.1	84.8	177.6	18	5	Pos	
Mannitol/Sorbitol	181.1	59	225	8	5	Neg	
Melatonin	233.2	174.1	105.2	12	5	Pos	
Mesoxalate	119.1	91.1	43.8	16	5	Pos	
Metanephrine	179.9	148.2	45.6	20	3	Pos	
Metanephrine/Selenomethionine	198.2	180.9	280	12	5	Pos	
Methionine	150.1	133	195.9	12	5	Pos	
Methionine sulfoxide (feature 1)	166.1	74	167	10	5	Pos	Removed after QC
Methionine sulfoxide (feature 2)	166.1	103	166.2	15	5	Pos	
Methylcrotonylcarnitine (C5:1)	244.1	85	105	18	5	Pos	
Methylcrotonylcarnitine (C5:1)	244	85.1	101.2	30	5	Pos	
Methylcysteine (feature 1)	136	119	207	12	5	Pos	
Methylcysteine (feature 2)	136	119	223	12	5	Pos	
Methylguanidine	74.2	42.2	181.9	10	5	Pos	
Methylhistidine (feature 1)	170.1	96	347.2	22	5	Pos	
Methylhistidine (feature 2)	170.1	96	320	22	5	Pos	
Methylmalonic acid	117	73.1	116.9	13	5	Neg	
Methylnicotinamide	137	94	165	20	5	Pos	
Monosaccharide	198	163	237	6	5	Pos	
Myo-inositol	179	59.8	226.6	10	5	Neg	
N-Acetyl-D-glucosamine	222	138	202.6	18	5	Pos	Removed after QC
N-Acetyl-DL-methionine	190.2	147.9	178.2	12	5	Pos	
N-Acetyl-L-alanine	130	88.2	117.6	12	5	Pos	
N-Acetyl-L-asparagine	173.1	154.9	209.9	8	5	Neg	
N-Acetyl-L-aspartic acid	174	88	207.1	12	5	Neg	

Table S1. Continued.

N-Acetyl-L-Cysteine	163.1	43.2	213.2	17	5	Pos	
N-Acetylasparagine	175.2	133	367.2	11	5	Pos	
N-Acetylaspartate/N-Amidino-L-aspartate	176.1	134	251.3	10	5	Pos	
N-Acetylglucosamine phosphate	300	79	169.8	34	5	Neg	Removed after QC
N-Acetylglutamic acid	190.2	130.1	327	12	5	Pos	
N-Acetylglutamine (feature 1)	189.1	130	339.6	17	5	Pos	detected RT: 326
N-Acetylglutamine (feature 2)	189.1	130	339.6	17	5	Pos	detected RT: 351
N-Acetylglycine	118.2	72.1	204.6	16	5	Pos	
N-acetylornithine	175.1	115.1	363	10	5	Pos	
N-Acetylserine	146	116	204	4	5	Neg	Removed after QC
N-Alpha-Acetyl-L-lysine	187.1	144.9	234	8	5	Neg	Removed after QC
N-carbamoyl-L-aspartate	175	132	277.8	13	5	Neg	
N-monomethylarginine (NMMA)	189.2	74	326.2	15	5	Pos	
N6-acetyl-L-lysine	189.1	126.1	343.8	10	5	Pos	
N6, N6, N6-Trimethyllysine (feature 1)	189.2	130.1	321	14	5	Pos	
N6, N6, N6-Trimethyllysine (feature 2)	189.2	130.1	343.8	14	5	Pos	
NAADP	745.1	604	70.7	20	5	Pos	Removed after QC
NADH	666.1	514	111	28	5	Pos	Removed after QC
NG,NG-Dimethylarginine	203	70	293.6	24	5	Pos	
Nicotinamide	123	79.9	210.8	18	5	Pos	
Nicotinamide riboside	255.1	56.9	94.1	24	5	Pos	
Nicotinamide ribotide	335	123	48	30	5	Pos	Removed after QC
Nicotinic acid	124	80.1	321.4	18	5	Pos	
Nitroarginine	220.1	59.1	180	35	5	Pos	
NMN (-H2O)	301.1	134.1	39.4	35	5	Pos	Removed after QC
Noradrenaline	170.2	93	321	26	5	Pos	
Norleucine	132.2	69.1	183.1	16	5	Pos	Removed after manual review
Norvaline	118.2	72.1	208	16	5	Pos	

Table S1. Continued.

O-Acetyl-L-serine	148.1	106	186	10	5	Pos	Removed after QC
O-succinyl-L-homoserine	220.1	102.1	210	11	5	Pos	Removed after QC
Octadecenoyl carnitine (C18:1)	426.3	85	67	15	5	Pos	
Octadiecadienoylcarnitine	424.3	85	58.9	32	5	Pos	
Octenoylcarnitine (C8:1)	286.1	85	73.6	24	5	Pos	
Orotate	155	111	186	10	5	Neg	Removed after QC
Orotidine-5-phosphate	367	323	46.9	18	5	Neg	
Orotidylic acid	367	97	65.1	10	5	Neg	Removed after QC
Oxalate	89	45	102	8	5	Neg	
Oxaloacetate	131	87	104.4	8	5	Neg	
Oxoguanine/3-Methoxytyramine	168	151	120	20	5	Pos	
Oxypurinol	153	136	67.2	20	5	Pos	
Palmitoylcarnitine	400.3	85	56.3	15	5	Pos	
Pantothenic acid (Vitamin B5)	218	146	90	21	5	Neg	Removed after QC
Paraxanthine	181.2	124.1	58	21	5	Pos	Removed after QC
Phenylacetylglycine	194.1	76	197.1	10	5	Pos	
Phenylalanine	166	120	168	10	5	Pos	
Phenylpyruvate	163	91	105.6	13	5	Neg	Removed after QC
Phospho(enol) pyruvic acid	167	79	120	10	5	Pos	Removed after QC
Phosphocreatine	212	43.9	201	24	5	Pos	
Phosphoglycerates	185	97	156	10	5	Neg	Removed after QC
Phosphorylcholine	183	125	87	23	5	Pos	
Pipecolic acid (feature 1)	138	84	174.6	18	5	Pos	
Pipecolic acid (feature 2)	138	84	202.8	18	5	Pos	
Progesterone	313.2	276.8	211.1	25	5	Neg	
Proline	116.1	70.1	214.1	13	5	Pos	
Propionylcarnitine (C3)	218.1	85	132.3	15	5	Pos	Removed after manual review
Purine	121.1	94	166.8	21	5	Pos	

Table S1. Continued.

Putrescine	89	72	296.1	12	5	Pos	Removed after manual review
Pyrazole	68	41.2	212.4	19	5	Pos	
Pyridoxamine (Vitamin B6)	169	152	200	11	5	Pos	
Pyridoxine (Vitamin B6)	170	134	39	18	5	Pos	
Pyrrolidinone	86.1	69.1	183.4	10	5	Pos	
Ribose	149	59.1	222	8	5	Neg	Removed after manual review
S-methyl ergothioneine	244.1	141	210	20	5	Pos	
S-methyl-5-thioadenosine	298	136	156.6	29	5	Pos	Removed after QC
S-Nitrosoglutathione	337.1	307.1	57.6	5	5	Pos	
Sarcosine	90	44.1	251.8	20	5	Pos	
Sedoheptulose 1,7-bisphosphate	369	166	73.4	20	5	Neg	Removed after QC
Serine	106	60	149.7	15	5	Pos	
Shikimate	173	93.1	69.2	18	5	Neg	
Spermidine	146.2	72	201.6	22	5	Pos	
Spermine	202.1	129.1	114	19	5	Pos	
Stearoyl-L-carnitine	428.4	85	53.4	15	5	Pos	
Succinate	116.9	73	104.4	10	5	Neg	
Succinylcarnitine (C4-DC)/3-hydroxyisovalerylcarnitine (C5-OH)	262	85.1	160.9	22	5	Pos	
Symmetrical dimethylarginine (SDMA)	203.2	171.9	293.4	9	5	Pos	
Taurine	124	80	245.8	18	5	Neg	Removed after QC
Taurochenodeoxycholic Acid (TCDCA)/Taurodeoxycholic Acid (TDCA)/Tauroursodeoxycholic acid (TUDCA) (feature 1)	498.3	498.3	124.2	20	5	Neg	detected RT: 40
Taurochenodeoxycholic Acid (TCDCA)/Taurodeoxycholic Acid (TDCA)/Tauroursodeoxycholic acid (TUDCA) (feature 2)	498.3	498.3	124.2	20	5	Neg	detected RT: 118; Removed after manual review

Table S1. Continued.

Taurochenodeoxycholic Acid (TCDCA)/Taurodeoxycholic Acid (TDCA)/Tauroursodeoxycholic acid (TUDCA) (feature 3)	498.3	498.3	126	20	5	Neg	
Taurocholate (TCA)	514.3	514.3	42.8	20	5	Neg	
Taurolithocholic acid (TLCA)	498.3	80	122.9	70	5	Neg	
Testosterone	289.1	109	42.6	22	5	Pos	Removed after QC
Tetrahydrofolate	446.1	430.1	37.8	11	5	Pos	Removed after QC
Theobromine	181.2	138	56.4	17	5	Pos	
Theophylline	181.1	124.1	57.6	19	5	Pos	Removed after QC
Thiamine (Vitamin B1)	265	122	170.5	19	5	Pos	
Thiourea	77	60	196.4	10	5	Pos	
Thymine	127.1	110	350.3	19	5	Pos	
Trans-4-hydroxyproline (feature 1)	132	85.9	175.1	14	5	Pos	
Trans-4-hydroxyproline (feature 2)	132	85.9	183.5	14	5	Pos	Removed after QC
Trigonelline	138.1	92.1	195	19	5	Pos	
Trimethylamine (TMA)	144.1	55.1	180	30	6	Pos	
Trimethylamine oxide (TMAO)	76	58	197.4	25	5	Pos	
Triuret (feature 1)	146.9	86.9	162	6	5	Pos	
Triuret (feature 2)	146.9	86.9	198	6	5	Pos	Removed after QC
Tryptophan	205.1	188.1	177.5	10	5	Pos	
Tyrosine	182	136	223.2	8	5	Pos	
Uracil	113	70	125	20	5	Pos	
Urea	61.1	44.2	105.2	25	5	Pos	
Urea/Ethanolamine	62.1	44.2	180	12	5	Pos	
Uric acid	167	123.9	204.5	13	3	Neg	
Uridine	245.1	113	127.2	8	5	Pos	
Valine	118	72	207.2	8	5	Pos	

Table S1. Continued.

Vitamin D2 (feature 1)	397.3	397.3	42.5	5	5	Pos	detected RT: 39
Vitamin D2 (feature 2)	397.3	397.3	42.5	5	5	Pos	detected RT: 45; Removed after QC
Vitamin D3	383.3	339.1	48	13	5	Neg	Removed after manual review
Vitamin K1	451.4	187	110.8	25	5	Pos	Removed after QC
Xanthine	151	108	123	16	5	Neg	
Xanthosine	285	153	165	10	5	Pos	
Xanthurenic acid	204	160	216	19	5	Neg	Removed after QC
**ISTD**							
7-HCO ISTD	408.1	97.2	42	32	5	Pos	
7,12-DHCO ISTD	425.2	398.3	42	15	5	Pos	
8- F2 isop ISTD	357.4	197.1	48	30	5	Neg	
ADMA ISTD	210.4	77.1	299.5	21	5	Pos	
Allaintoin ISTD	162.9	117.8	166	20	5	Neg	
Arginine ISTD	185.1	122.1	366	25	5	Pos	
Aspartic acid ISTD	139.1	92.1	179.8	12	5	Pos	
BAIBA ISTD	107.2	89	234	5	5	Pos	
butyryl carnithine ISTD	235.1	84.9	108	20	5	Pos	
CDCADCA-UDCA-D4	395.3	395.3	48	30	5	Neg	
Creatinine ISTD	117.1	47.1	149.7	19	5	Pos	
Ergo ISTD	239.3	127	234	20	5	Pos	
GCA-D4 ISTD	468.3	74	96	70	5	Neg	
GCDCA-D4 ISTD	452.3	74	58.8	72	5	Neg	
glutamic acid ISTD	154.1	89.1	277.8	10	7	Pos	
glutamine ISTD	152.1	88.1	258	15	5	Pos	
Hercynine ISTD	207.2	163.2	288	9	4	Pos	
Histidine ISTD	165.2	118.2	354	15	5	Pos	
Homocysteine ISTD	140.2	94.2	362.4	15	5	Pos	

Table S1. Continued.

Isoleucine ISTD	139	74.1	180	20	5	Pos
Isovalerylcarnitine ISTD	255.2	85.1	91.8	20	5	Pos
LCA D4	379.3	379.3	45.6	30	5	Neg
leucine ISTD	138.1	91.2	195.6	10	5	Pos
Lysine ISTD	155.2	90.1	36.72	18	7	Pos
Methionine ISTD	156.2	109	194.4	15	5	Pos
Phenylalanine ISTD	176.1	129.1	168.6	10	5	Pos
Proline ISTD	122.1	75.1	208.8	14	5	Pos
Propionylcarnitine ISTD	221.2	85.1	120	20	5	Pos
Serine ISTD	110.2	63.1	279.6	8	3	Pos
Spermidine ISTD	150.2	76.1	201.6	15	5	Pos
TDCA ISTD	502.2	124.2	103.2	57	7	Neg
Threonine ISTD	125.1	78.1	259	10	7	Pos
Tyrosine ISTD	192.1	98.1	224.4	30	7	Pos
UA ISTD	169	125.1	214.8	13	3	Neg
Valine ISTD	123.1	76.1	208.8	15	5	Pos
Xanthine ISTD	153.1	109.1	126	18	5	Neg

Table S2: Detailed composition of the test meals (TM) and standardized meals (SM).

	Quantity (g)	Energy (kcal)	Protein (g)	Fat (g)	CHO (g)	Fiber (g)	Available CHO (g)
**TM High GI (92)**							
Glutinous rice	100.7	356.6	7.7	2.3	76.1	1.0	75.1
Chicken seasoning	2.0	4.5	0.3	0.1	0.7	0	0.7
Green leafy vegetables	20.0	6.9	0.6	0.4	0.3	0.1	0.2
**TOTAL**	**122.7**	**368.0**	**8.5**	**2.7**	**77.1**	**1.1**	**76.0**
**TM Low GI (55)**							
Parboiled Basmati rice	99.8	349.1	9.4	0.7	76.4	1.4	75.0
Chicken seasoning	2.0	4.5	0.3	0.1	0.7	0	0.7
Green leafy vegetables	20.0	6.9	0.6	0.4	0.3	0.1	0.2
**TOTAL**	**121.8**	**360.5**	**10.2**	**1.1**	**77.4**	**1.5**	**75.9**
**SM**							
Shrimp wanton soup	145.0	132.0	8.1	3.0	18.0	2.8	15.2
Soya milk	300.0	162.0	6.3	3.3	26.4	1.8	24.6
Soybeans- based snack	27.0	111.0	4.3	5.5	11.0	2.5	8.5
Almond nuts	40.0	233.0	9.2	19.5	5.3	4.3	1.1
Banana	125.0	110.0	1.0	0.0	30.0	3.0	27.0
**TOTAL**	**637.0**	**748.0**	**28.9**	**31.3**	**90.7**	**14.4**	**76.4**

The meals immediately prior to the test meals were standardized, i.e. the dinner prior to the breakfast test sessions and the lunch prior to the dinner test sessions. CHO: carbohydrate; GI: glycemic index; TM: test meal; SM: standard meal.

Figure S2. Level-time line plots of the metabolites with significant *p*-values (HI-DI: high-GI dinner; LO-DI: low-GI dinner; HI-BR: high-GI breakfast; LO-BR: low-GI breakfast).

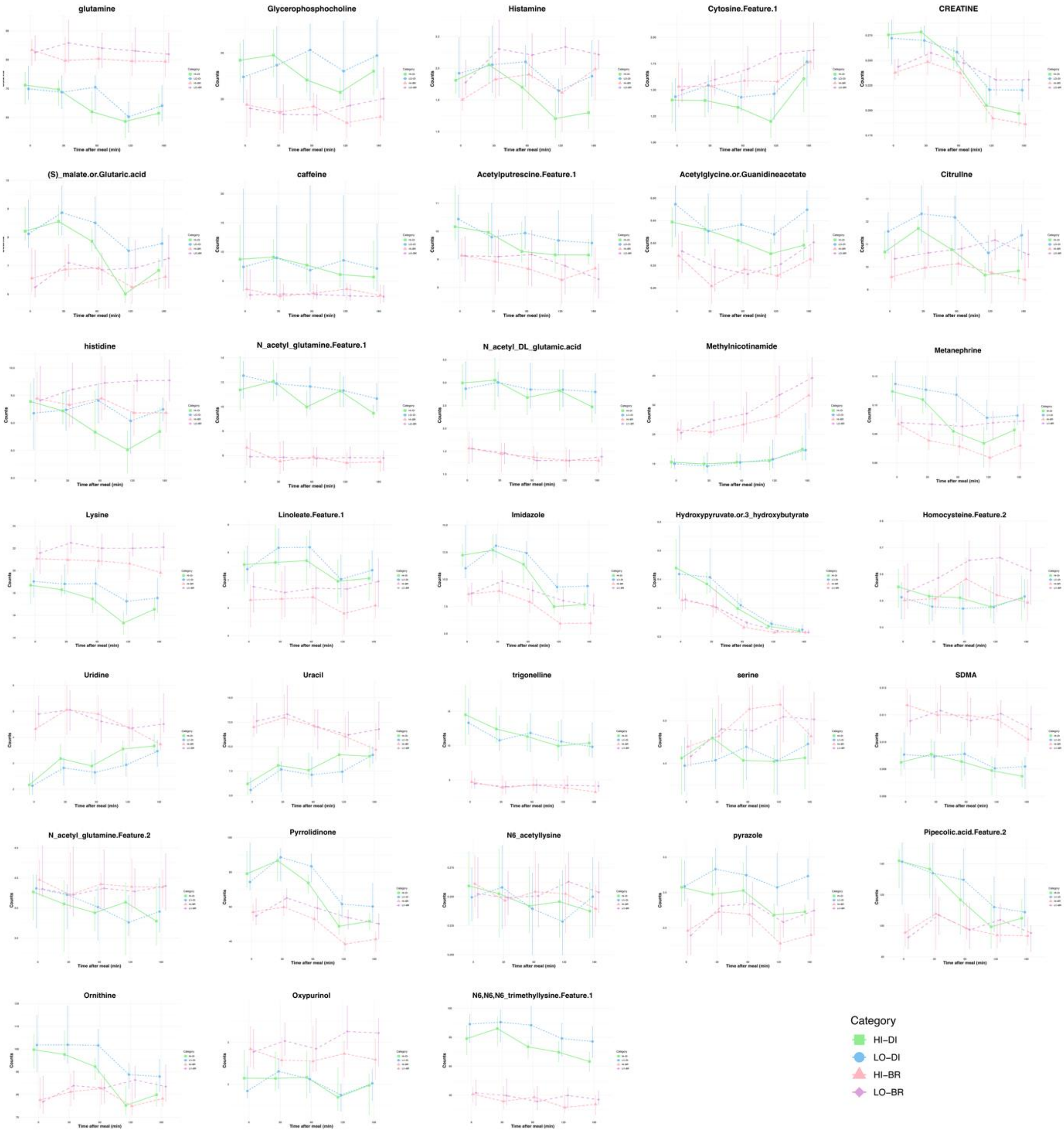

Figure S3. Sample pathway map of Arginine-NO pathway.

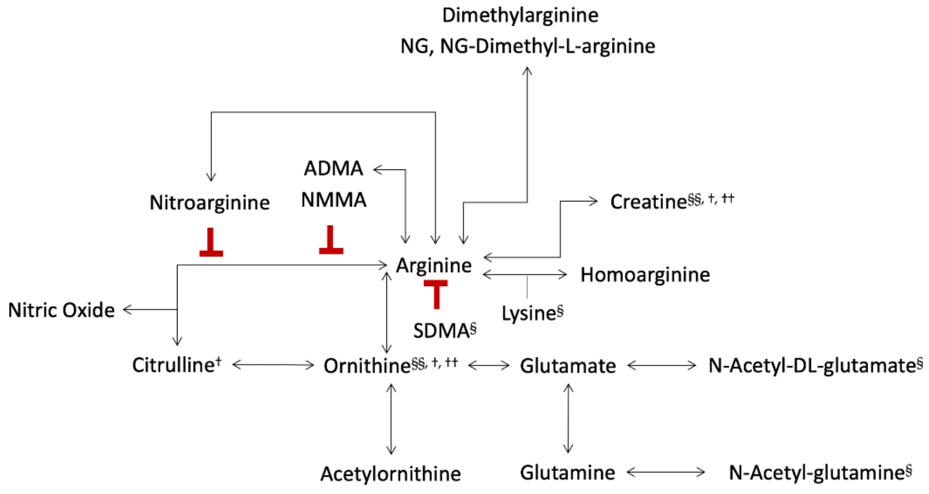

§: the metabolite had significantly different AUC in DI vs BR; §§: the metabolite had significantly different δAUC in DI vs BR; †: the metabolite had significantly different AUC in HI vs LO; ††: the metabolite had significantly different δAUC in HI vs LO.

1. Teo, G.; Chew, W.S.; Burla, B.J.; Herr, D.; Tai, E.S.; Wenk, M.R.; Torta, F.; Choi, H. MRMkit: Automated Data Processing for Large-Scale Targeted Metabolomics Analysis. *Anal Chem* **2020**, *92*, 13677-13682, doi:10.1021/acs.analchem.0c03060.
2. Broadhurst, D.; Goodacre, R.; Reinke, S.N.; Kuligowski, J.; Wilson, I.D.; Lewis, M.R.; Dunn, W.B. Guidelines and considerations for the use of system suitability and quality control samples in mass spectrometry assays applied in untargeted clinical metabolomic studies. *Metabolomics* **2018**, *14*, 72, doi:10.1007/s11306-018-1367-3.
